# Muscle-specific regulation of right ventricular transcriptional responses to chronic hypoxia-induced hypertrophy by the muscle ring finger-1 (MuRF1) ubiquitin ligase in mice

**DOI:** 10.1186/s12881-018-0670-1

**Published:** 2018-09-21

**Authors:** Robert H. Oakley, Matthew J. Campen, Michael L. Paffett, Xin Chen, Zhongjing Wang, Traci L. Parry, Carolyn Hillhouse, John A. Cidlowski, Monte S. Willis

**Affiliations:** 1 Department of Health and Human Services, Signal Transduction Laboratory, National Institute of Environmental Health Sciences, National Institutes of Health, Research Triangle Park, NC USA; 2 Department of Pharmaceutical Sciences, College of Pharmacy, University of New Mexico Health Sciences Center, Albuquerque, NM USA; 3 McAllister Heart Institute, University of North Carolina, Chapel Hill, NC USA; 4 Department of Surgery, University of North Carolina, Chapel Hill, NC USA; 5 Department of Pathology & Laboratory Medicine, Indiana University School of Medicine, 635 Barnhill Drive, Van Nuys MS 5067, Indianapolis, IN 46202 USA; 6 Krannert Institute of Cardiology and Division of Cardiology, Department of Internal Medicine, Indiana University School of Medicine, Indianapolis, IN USA

**Keywords:** MuRF1, Hypoxia, Right heart failure, Gene expression, Microarray

## Abstract

**Background:**

We recently identified a role for the muscle-specific ubiquitin ligase MuRF1 in right-sided heart failure secondary to pulmonary hypertension induced by chronic hypoxia (CH). MuRF1−/− mice exposed to CH are resistant to right ventricular (RV) dysfunction whereas MuRF1 Tg + mice exhibit impaired function indicative of heart failure. The present study was undertaken to understand the underlying transcriptional alterations in the RV of MuRF1−/− and MuRF1 Tg + mice.

**Methods:**

Microarray analysis was performed on RNA isolated from the RV of MuRF1−/−, MuRF1 Tg+, and wild-type control mice exposed to CH.

**Results:**

MuRF1−/− RV differentially expressed 590 genes in response to CH. Analysis of the top 66 genes (> 2-fold or < − 2-fold) revealed significant associations with oxidoreductase, transcription regulation, and transmembrane component annotations. The significant genes had promoters enriched for HOXD12, HOXC13, and RREB-1 protein transcription factor binding sites. MuRF1 Tg + RV differentially expressed 150 genes in response to CH. Analysis of the top 45 genes (> 3-fold or < − 3-fold) revealed significant associations with oxidoreductase-metabolic, glycoprotein-transmembrane-integral proteins, and alternative splicing/splice variant annotations. The significant genes were enriched for promoters with ZIC1 protein transcription factor binding sites.

**Conclusions:**

The differentially expressed genes in MuRF1−/− and MuRF1 Tg + RV after CH have common functional annotations related to oxidoreductase (including antioxidant) and transmembrane component functions. Moreover, the functionally-enhanced MuRF1−/− hearts regulate genes related to transcription, homeobox proteins, and kinases/phosphorylation. These studies also reveal potential indirect effects of MuRF1 through regulating Rreb-1, and they reveal mechanisms by which MuRF1 may transcriptionally regulate anti-oxidant systems in the face of right heart failure.

**Electronic supplementary material:**

The online version of this article (10.1186/s12881-018-0670-1) contains supplementary material, which is available to authorized users.

## Background

Right ventricular heart failure is the most frequent cause of death in patients with pulmonary arterial hypertrophy (PAH) [1, 2]. In general, chronic hypoxia (CH) in the lungs induces an elevation in pulmonary artery pressures, increasing the afterload in the right ventricle. In response, right ventricular (RV) remodeling results, possibly including maladaptive hypertrophy. Recent reviews have outlined the molecular and cellular drivers of right ventricular remodeling in chronic pressure overload, including the induction of cardiomyocyte cell death, increases in collagen synthesis and decreases in collagen degradation, decreases in capillary density and a metabolic shift to glycolysis [3]. Rho-kinase [4], adrenergic signaling [5] and altered angiogenesis have been reported to play roles in the pathogenesis of RV remodeling at the molecular level. Maladaptive RV hypertrophy characteristically exhibits a shift to aerobic glycolysis mediated by FOXO1 [6] and c-Myc [7]. Beyond this, little is currently known about the molecular regulation of these underlying mechanisms that occur during PAH-induced RV heart failure.

The muscle-specific ubiquitin ligase Muscle Ring Finger-1 (MuRF1) regulates both physiological and pathological hypertrophy and metabolism by interacting with specific transcription factors that mediate these processes. MuRF1 interacts with and inhibits serum response factor [8], which is upregulated in pathological pressure overload-induced hypertrophy. As such, MuRF1−/− mice exhibited exaggerated cardiac hypertrophy after transaortic constriction in vivo [8]. MuRF1 interacts with the c-Jun transcription factor in a proteasome-dependent manner to inhibit downstream AP1 activity, implicated in inhibiting IGF-1 signaling [9]. Consistent with this, MuRF1−/− mice voluntarily ran farther and faster without training, improving even more after training compared to wild-type mice [9]. The mechanisms by which cardiomyocyte MuRF1 inhibits the PPARalpha and TRalpha nuclear receptors appear to involve multi-mono-ubiquitination, resulting in changes in metabolism in vivo [9, 10].

Given transcriptional regulation by MuRF1 in cardiomyocytes, we recently hypothesized a role for MuRF1 in the pathophysiology of right ventricle heart failure [11]. We exposed MuRF1−/− and cardiomyocyte-specific MuRF1 transgenic (MuRF1 Tg+) mice to CH, resulting in an elevated pulmonary artery pressure/resistance and measured functional and morphological responses [11]. We found that MuRF1−/− mice exhibited greater RV growth compared to wild-type mice but were resistant to CH-induced changes in ejection fraction. As such, the overall output in MuRF1−/− hearts provided significantly increased perfusion to skeletal muscle compared to sibling wild-type mice [11]. In contrast, MuRF1 Tg + mice exhibited similar patterns in RV weight compared to wild-type mice in response to CH, but there was a reduced ejection fraction, indicative of a maladaptive dilated phenotype (heart failure), consistent with the significant decreases in skeletal muscle perfusion [11]. The present study was undertaken to understand the underlying transcriptional alterations that occur during CH in the right ventricle of MuRF1−/− and MuRF1 Tg + mice using microarray analysis.

## Methods

### MuRF1−/− and αMHC transgenic MuRF1 mice and the hypoxia-induced pulmonary hypertension model of right sided heart failure

MuRF1−/− and MuRF1 Tg mice and their strain-match wild-type controls (previously described [8, 12]) were shipped to the University of New Mexico. Following a week-long quarantine, mice maintained in standard shoebox cages were moved to either control or normobaric hypoxia chambers with continuous access to food and water ad libitum throughout. The hypoxia chamber was set at 10.0% oxygen (partial pressure of oxygen approximately 65 mmHg in Albuquerque, NM) and monitored both by a digital feedback control system (Biospherix, Parish, NY) as well as by a secondary O_2_/CO_2_ monitor (O_2_Cap, OxiGraf, Inc.; Mountain View, CA), as recently described [11]. Hypoxia exposure lasted 3 weeks with twice-weekly cage changes and standard 12 h light:dark cycle. Procedures were conducted under full isoflurane anesthesia to minimize or eliminate risk of pain and discomfort. Mice were anesthetized with isoflurane and were euthanized by exsanguination, followed by rapid removal of the heart into ice-cold phosphate-buffered saline. The RV free wall was dissected away from the rest of the heart, as previously described [13]. Mice were bred at the University of North Carolina at Chapel Hill and all the hypoxia procedures were conducted at the University of New Mexico with full approval of the UNC-Chapel Hill and University of New Mexico Institutional Animal Care and Use Committees and were carried out in compliance with the National Institutes of Health Guide for the Care and Use of Laboratory Animals.

### Microarray assay and statistical analysis

Microarrays were performed on RNA isolated from RV tissue from animals challenged with hypoxia. At least three independent samples were assayed from each of the four experimental groups MuRF1−/− (*N* = 5), its strain matched wild-type MuRF1+/+ (*N* = 6), MuRF1 Tg + (*N* = 3), and its strain-matched wild type (N = 3). Gene expression assay was performed using Agilent Whole Mouse 4 × 44 k multiplex format oligo arrays (014868, Agilent Technologies, Santa Clara, CA) following the Agilent 1-color microarray-based gene expression analysis protocol. Data were obtained using Agilent Feature Extraction software (version 12), using the 1-color defaults for all parameters. The Agilent Feature Extraction software performed error remodeling, adjusting for additive and multiplicative noise. To determine differentially expressed genes between groups, an ANOVA with multiple test correction (FDR q-value) was performed using Partek Genomics Suite software (version 6.6). Only one gene for MuRF1−/− and 6 genes for MuRF1 Tg + passed the FDR q-value < 0.05 cutoff; therefore, the set of differentially expressed genes was expanded to include genes from the ANOVA analysis with an unadjusted *p*-value < 0.01 (see Additional file 1: Table S1 and Additional file 2: Table S2). To minimize inclusion of false positives, genes with fold changes greater than 2 and less than − 2 (for MuRF1−/− vs. MuRF1+/+) or greater than 3 and less than − 3 (for MuRF1 Tg + vs. MuRF1+/+) were selected for downstream bioinformatic analyses. The differential expression of a subset of selected genes was confirmed by RT-PCR. The MAIME-compliant data were submitted to Gene Expression Omnibus and assigned the accession #GSE82345 (http://www.ncbi.nlm.nih.gov/geo/query/acc.cgi?token=sfavmocqtrupnan&acc=GSE82345, currently embargoed/not publicly available).

### Bioinformatics resources and analytic tools used and retrieval of public microarray data

TRANSFAC® FMatch was used to analyze transcription factors (https://portal.biobase-international.com, GeneExplan GmbH). The TRANSFAC MATRIX TABLE, Release 2016.2 was used as the matrix library. To minimize false-positive matches, the matrix file vertebrate_non_redundant_minFP was used for transcription factor binding sites prediction, using cut-off criteria to minimize false-positives, and a *p*-value threshold set at 0.01. The significant gene sets for the MuRF1−/− (66 unique genes) and MuRF1 Tg + (45 unique genes) were used in the STRING analysis. Duke Gather Literature Net. Genes were analyzed at http://changlab.uth.tmc.edu/gather/ via Literature Net. STRING database. The STRING database (STRING Consortium, Swiss Institute of Bioinformatics, Zurich, Switzerland) of known and predicted protein-protein interactions (https://string-db.org/) includes direct (physical) and indirect (functional) associations aggregated from other (primary) databases [14–16]. The significant gene sets for the MuRF1−/− (66 unique genes) and MuRF1 Tg + (45 unique genes) were used in the STRING analysis. The MuRF1−/− significant networks contained 26 nodes (vs. expected 3 nodes), 74 edges, average node degree 5.69, with a clustering coefficient of 0.735. The MuRF1 Tg + significant networks contained 26 nodes, 75 edges (28 expected), average node degree 5.77 and a clustering coefficient of 0.696. The Database for Annotation, Visualization and Integrated Discovery (DAVID) v6.8 Functional Annotation Bioinformatics database was used to analyze differentially expressed genes (https://david.ncifcrf.gov/) [17, 18]. Analysis of gene lists (gene symbols) were uploaded using the gene accession conversion tool and analyzed using Disease (OMIM_Disease), Functional Categories (COG_Ontology, UP_Keywords, UP_SEQ_FEATURE) Gene Ontology (GOTERM_BP_DIRECT, GOTERM_CC_DIRECT, GOTERM_MF_DIRECT), Pathways (BBID, BIOCARTA, KEGG PATHWAY) and Protein Domains (INTERPRO, PIR_SUPERFAMILY, SMART). Microarray data retrieval and experimental design. The SuHx model of severe angio-obliterative pulmonary artery hypertension and RV failure was used for male Sprague-Dawley rats receiving a single subcutaneous injection of SU5416 (20 mg/kg) prior to 4 weeks of hypoxia [2]. Microarray analysis (Agilent whole rat genome 4 × 44 k microarray slide, Agilent Technologies, Wilmington, DE) was performed on snap-frozen right heart tissues [2]. Study datasets were retrieved from the publicly available NCBI Gene Expression Omnibus (GEO Dataset_GSE42579). Microarray replicates included 12 hypoxia and 8 normoxia from 6 biological replicates per group (hypoxia, normoxia). The GEO2R tool was used to download the ID, adj. *p* value, *p* value, t value, B value, log fold change (logFC), gene symbol, gene title, and gene ID of the differentially expressed genes for further analysis and comparison.

### RT qPCR analysis of mRNA expression

Total RNA was isolated using RNeasy Mini kits (QIAGEN, Valencia, CA) according to the manufacturer’s protocols. Determination of gene expression was performed using either a two-step RT-qPCR reaction [8, 19] or a one-step RT-qPCR reaction, as previously described [20]. For the two-step reaction, cDNA was made using iScript cDNA Synthesis kit, according to the manufacturer’s protocol (BioRad, Hercules, CA, Cat# 1708890). One microliter of the resulting cDNA product was then amplified in a 20-μl final volume using the TaqMan® Universal PCR Master Mix. Each reaction included 1 μl of mouse specific TaqMan probes for MuRF1 (Mm01185221_m1), Atp2b2 (Mm00437640_m1), Elovl7 (Mm00512434_m1), Gbp3 (Mm00497606_m1), Cxcl9 (Mm00434946), Myl1 (Mm00659043_m1), Casq1 (Mm00486725_m1), GAPDH (glyceraldehyde-3-phosphate dehydrogenase, Probe Mm99999915_g1), or 18S (Hs99999901_s1) in triplicate (ThermoFisher Scientific, Inc., Waltham, MA). The relative expression of mRNA (ΔΔCT algorithm) was determined using GAPDH or 18S as an internal sample loading control.

### Statistical analysis

For qRT-PCR analyses, Student’s t-test was performed to determine significant differences between groups. All statistics were performed using GraphPad Prism (La Jolla, CA, Version 7.01) with *p* values < 0.05 considered statistically significant.

## Results

**Fig. 1:**
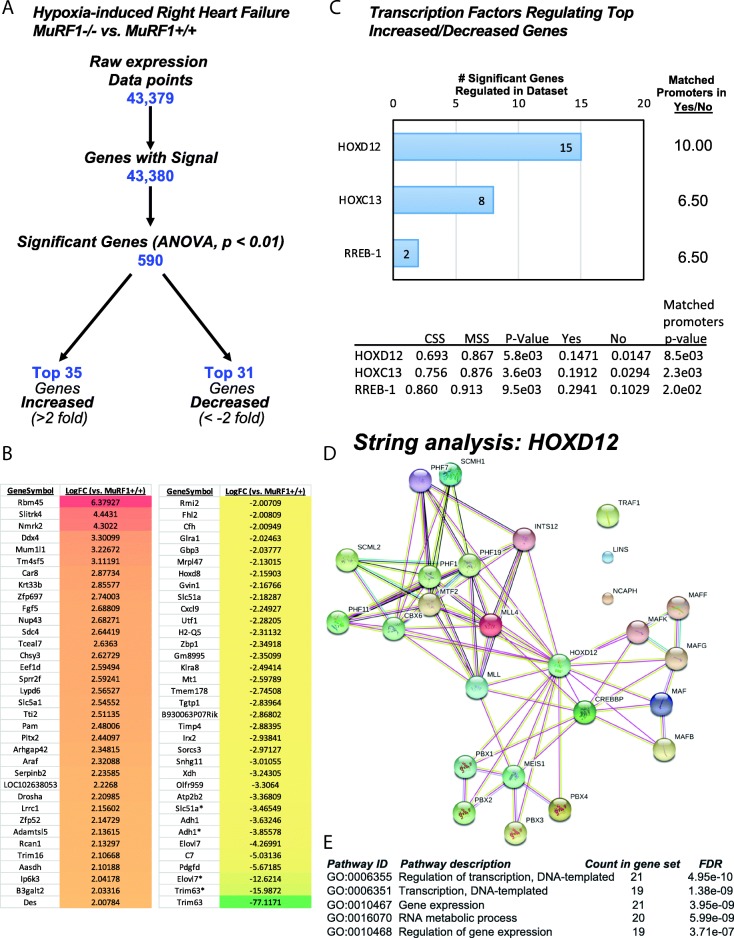
Differential gene expression of MuRF1−/− right ventricle after three weeks CH exposure. **a** Summary of how differentially expressed genes in hypoxia-challenged MuRF1−/− RV tissue compared to MuRF1+/+ were identified. **b** Top 35 increased unique genes (> 2-fold) and top 31 decreased unique genes (<− 2-fold) compared to MuRF1+/+ hearts. Asterisk (*) designates same/related sequence for duplicate gene name. **c** Identification of transcription factors with binding sites of top 35 increased and top 31 decreased genes using TRANSFAC® FMatch. The number of genes in dataset and enrichment (sites/sequence ratio) for each transcription factor is calculated to the right. **d** STRING analysis of differentially expressed genes for known interactions and **e** STRING analysis pathway ID statistics. MuRF1−/−: *N* = 5 biological replicates; MuRF1+/+: *N* = 6 biological replicates

To understand MuRF1’s role in the pathophysiology of CH-induced right heart failure, we performed microarray analysis on right ventricle tissue from MuRF1−/− and MuRF1 Tg + mice (and sibling wild-type controls) after three weeks of CH. Analysis of MuRF1−/− and MuRF1+/+ RV tissue by microarray analysis identified 590 genes to be significantly different from wild-type controls (ANOVA, *p* < 0.01) (Fig. 1a, Additional file 1: Table S1). We identified the top 35 unique genes increased (> 2-fold) and top 31 unique genes decreased (<− 2-fold) (Fig. 1b) and analyzed them for common transcription factors that may be responsible for the differential regulation of the MuRF1−/− mice using TRANSFAC® FMatch Analysis. We identified three (3) transcription factors that were enriched in significantly altered gene promoters in MuRF1−/− hypoxia-challenged RV compared to wild-type controls (left, Fig. 1c). These transcription factors are listed by the number of the binding sites found in the transcription factor sequences (sites/sequence ratio) reflecting how common these sites were for each transcription factor. The Homeobox D12 (HOXD12), Homeobox C13 (HOXC13), and the Ras Responsive Element Binding Protein 1 (RREB-1) were enriched 10-, 6.5- and 6.5-fold, respectively (Fig. 1c). Of the 66 significant genes used in the FMatch analysis, 15, 8 and 2 of these genes were associated with HOXD12, HOXC13 and RREB-1, respectively (Fig. 1c).

To understand how the differentially expressed genes in the MuRF1−/− right ventricle are known to interact, we performed a STRING analysis to identify relationships that had been reported between these gene products in the literature (Fig. 1d). We identified significantly more interactions than would be expected by chance. Specifically, the network had 26 modes and 74 edges compared to the expected 3 edges due to chance. The functional enrichments identified by STRING Gene Ontology (GO) included the regulation of transcription, gene expression and RNA metabolic processes (Fig. 1e). Overall, HOXD12 had the most interactions (20), while MTF2 and MLL4 had 9 and INTS12 had 6, demonstrating the importance of HOXD12 among these proteins based on our current knowledge of these genes.

**Fig. 2:**
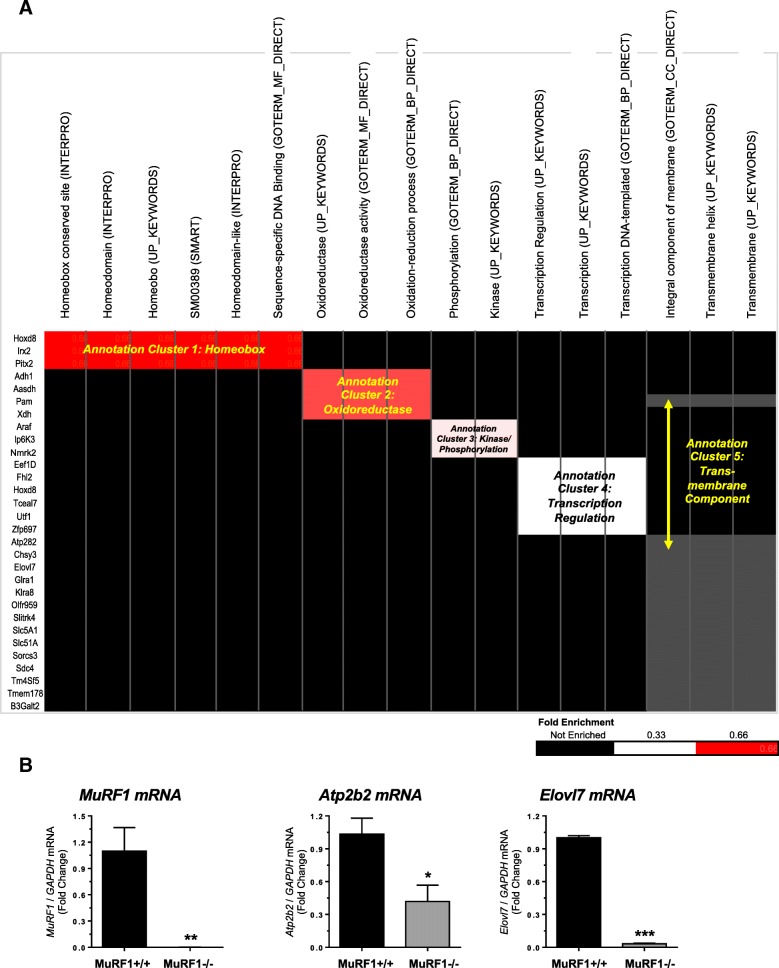
Functional annotation of differentially expressed genes in MuRF1−/− right ventricle after three weeks CH exposure. **a.** Hypercluster analysis of differentially expressed MuRF1−/− genes in the right ventricle after hypoxia challenge using the DAVID v6.8 Functional Annotation Bioinformatics database. Differentially expressed gene symbols are on the left, functional categories along the top (database source in parentheses). The cluster fold enrichment is color-coded (key in lower right). **b.** Reverse transcriptase quantitative PCR analysis of MuRF1−/− hearts of differentially expressed genes *MuRF1, Atp2b2*, and *Elovl7* mRNA in the right ventricle

To determine the functional significance of the 66 differentially expressed genes from the MuRF1−/− right ventricle in more detail, we performed functional annotations using both STRING and DAVID. STRING analysis of functional categories identified regulation of transcription, gene expression, and RNA metabolism as the significant pathways with low false discovery rates (Fig. 1e). DAVID analysis detailed these functions, identifying five functional clusters across several databases (Fig. 2a). Functional annotation identified 30 genes (Fig. 2a, far left) that exhibited reduced enrichment in five clusters, including Homeobox-related genes (cluster 1), genes involved in oxidoreductase (cluster 2), genes involved in phosphorylation/kinase activity (cluster 3), genes involved in the regulation of transcription (cluster 4), and genes involved in the transmembrane components (cluster 5) (Fig. 2a). We validated the differential expression of several genes in the MuRF1−/− right ventricle after CH, including *MuRF1*, *Atp2b2*, *Elovl7*, *Gbp3* and *Cxcl9 m*RNA by RT-qPCR analysis (Fig. 2b and Additional file 3: Figure S1). Together, these results demonstrate that 3 weeks of CH-induced changes in the MuRF1−/− right ventricle to alter functions associated with homeobox proteins (associated with gene expression), oxidoreductase processes, kinases/phosphorylation, transcriptional regulation and transmembrane components.

**Fig. 3:**
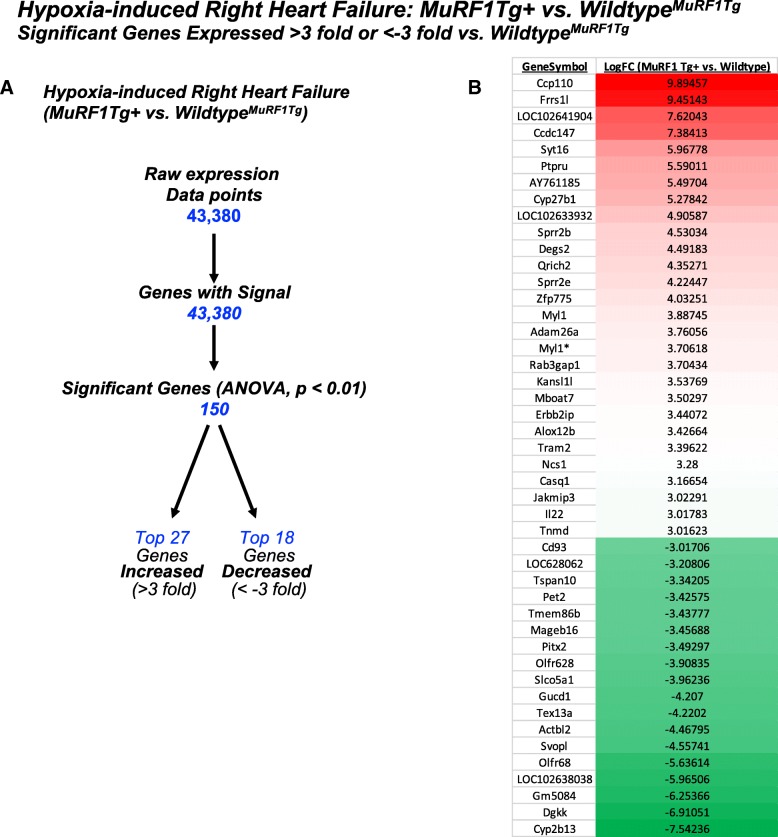
Differential gene expression of MuRF1 Tg + right ventricle after three weeks CH exposure. **a.** Summary of how differentially expressed genes in hypoxia-challenged MuRF1Tg + right ventricles were identified. **b.** Top 27 increased unique genes (> 3-fold) and top 18 decreased unique genes (<− 3-fold) compared to wild-type^MuRF1Tg+^ RV tissue. Asterisk (*) designates transcript variant for duplicate gene name. MuRF1 Tg+: *N* = 3; strain-matched wild type: *N* = 3

**Fig. 4:**
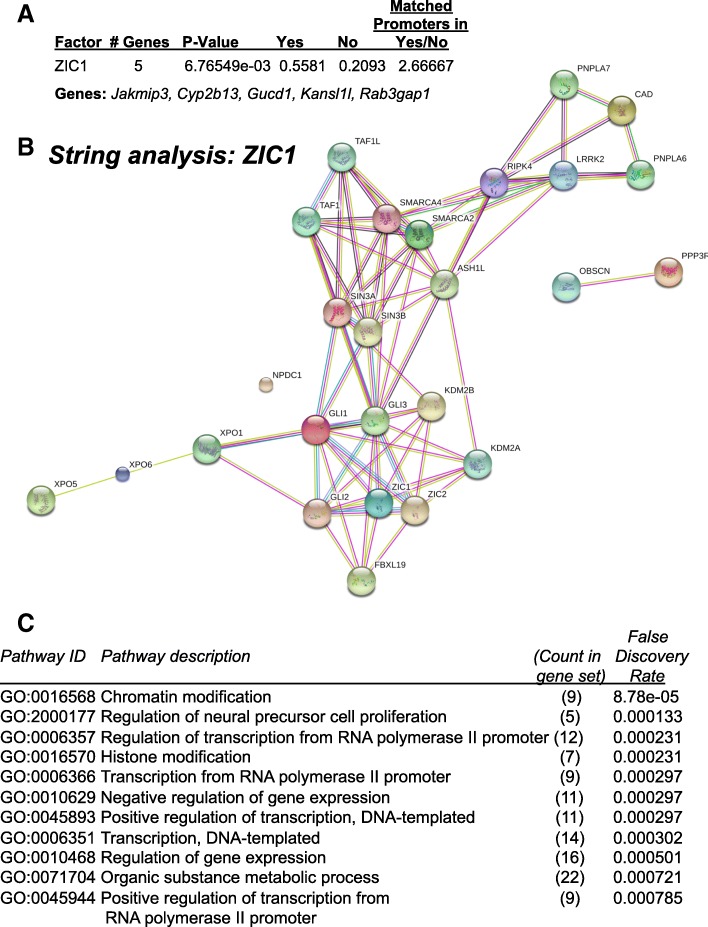
Functional annotation of differentially expressed genes in MuRF1 Tg + right ventricles after three weeks CH exposure. **a.** Identification of ZIC1 protein binding sites based on binding sites in 5 of the differentially expressed genes in MuRF1 Tg + heart (*Jakmip3, Cyp2b13, Gucd1, Kansl1l, Rab3gap1*). The number of genes in dataset and enrichment (sites/sequence ratio) for each transcription factor is calculated to the right. **b.** STRING analysis of the top 27 genes increased (> 3-fold) and top 18 genes decreased (<− 3-fold) listed in Fig. 3b. **c.** Functional annotation of differentially expressed MuRF1 Tg + right ventricle by STRING analysis

Analysis of MuRF1 Tg + and MuRF1+/+ RV tissue by microarray analysis identified 150 genes as significantly different from wild-type controls (ANOVA, *p* < 0.01) (Fig. 3a, Additional file 2: Table S2). We identified the top 27 unique genes increased (> 3-fold) and top 18 unique genes decreased (<− 3-fold) (Fig. 3b) so that we could identify the common transcription factors that may be responsible for the differential regulation of MuRF1Tg + mice using TRANSFAC® FMatch Analysis. Analysis of these 45 genes identified that they were enriched with promoter regions recognized by the ZIC1 (ZInc finger protein of the Cerebellum (Zic) Family Member 1) protein transcription factor in MuRF1Tg + − hypoxia-challenged RV compared to wild-type controls (Fig. 4a). The 2.6-fold enrichment in ZIC1 protein promoter sites (*p* = 6.76549e-03) was based on the significantly altered genes *Jakmip3* (Janus Kinase and Microtubule Interacting Protein 3), *Cyp2b13* (Cytochrome P450 Family 2 Subfamily B Member 6), *Gucd1* (Guanylyl Cyclase Domain Containing 1), *Kansl1l* (KAT8 Regulatory NSL Complex Subunit 1 Like) and *Rab3gap1* (RAB3 GTPase Activating Protein Catalytic Subunit 1) genes (Fig. 4a).

To identify how the genes that were differentially expressed in the MuRF1 Tg + right ventricle compared to wild-type controls after three weeks of CH, we performed a STRING analysis to identify relationships that had been reported between these gene products in the literature (Fig. 4b). Specifically, the network had 26 modes and 75 edges compared to the expected 28 edges due to chance (PPI enrichment *p*-value = 3.27e-13). The functional enrichments identified by STRING Gene Ontology (GO) included chromatin modification, histone modification and regulation of gene expression among other related functions (Fig. 4c). Overall, ZIC1 protein had 6 interactions across the STRING network and GL1 and GL13 had 9 and 13 interactions with the network, respectively, demonstrating the importance of ZIC1 within these proteins in the literature.

**Fig. 5:**
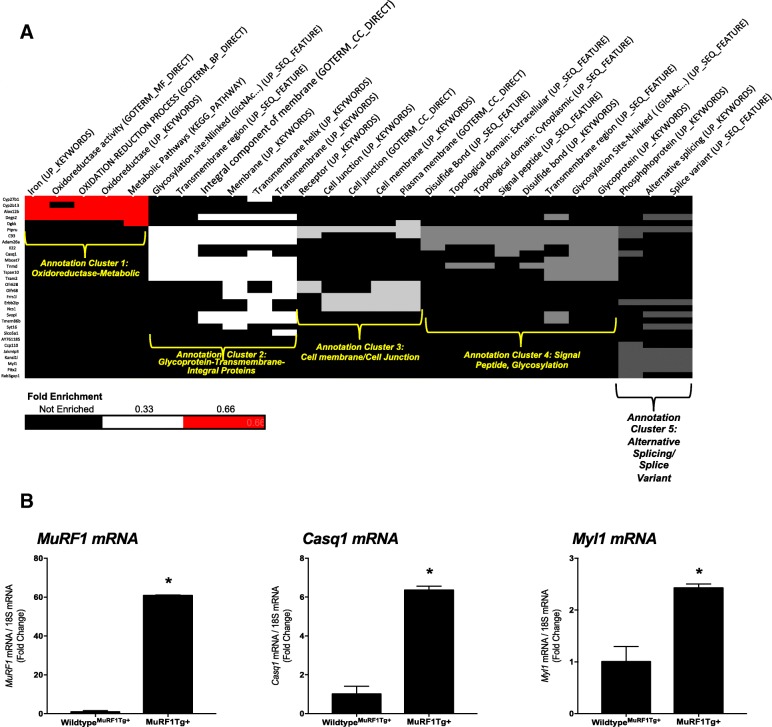
Functional annotation of differentially expressed genes in MuRF1 Tg + hearts compared to wild-type controls. **a.** Hypercluster analysis of differentially expressed MuRF1−/− genes in the right ventricle after hypoxia challenge using the DAVID v6.8 Functional Annotation Bioinformatics database. Differentially expressed gene symbols are on the left, functional categories along the top (database source in parentheses). The cluster fold enrichment is color-coded (key in lower left). **b.** Reverse transcriptase quantitative PCR analysis of MuRF1−/− hearts of differentially expressed genes *MuRF1, Casq1*, and *Myl1* mRNA in the right ventricle

We analyzed the 45 differentially expressed genes from the MuRF1 Tg + right ventricle after 3 weeks of CH using DAVID analysis to understand the biological relevance of the gene set. We found five clusters, including oxidoreductase-metabolic (cluster 1), glycoprotein-transmembrane-integral proteins (cluster 2), cell membrane/cell junction (cluster 3), signal peptide, glycosylation (cluster 4) and alternative splicing/splice variant (cluster 5) (Fig. 5a). We validated the differential expression of several genes in the MuRF1 Tg + right ventricle after CH, including *MuRF1*, *Casq1* and *Myl1* mRNA by RT-qPCR analysis (Fig. 5b). Analysis of the 45 differentially expressed genes from the MuRF1 Tg + right ventricle for their relationships in the literature [21] identified 12 groups, including links with PPAR binding protein (PPARBP), regulators of G-protein signaling (RGS5, RGS8, RGS12, RGS14, RGS20) and v-ros UR2 sarcoma virus oncogene (ROS1) (Additional file 4: Figure S2).

Comparison of the functional categories identified in the CH-challenged MuRF1−/− right ventricles (vs. wild type) (Fig. 2a) and the MuRF1 Tg + right ventricles (vs. wild type) (Fig. 5a) demonstrates overlapping and unique functional regulation in each model (Table 1). Both the MuRF1−/− and MuRF1 Tg + hearts have differential regulation of oxidoreductase functions and transmembrane component proteins (Table 1). The MuRF1−/− model uniquely regulated genes associated with homeobox proteins, kinase/phosphorylation, and transcriptional regulation (Table 1, left column) while the MuRF1 Tg + model uniquely regulated genes associated with signal peptide/glycosylation and alternative splicing (Table 1, right column).

**Table 1:**
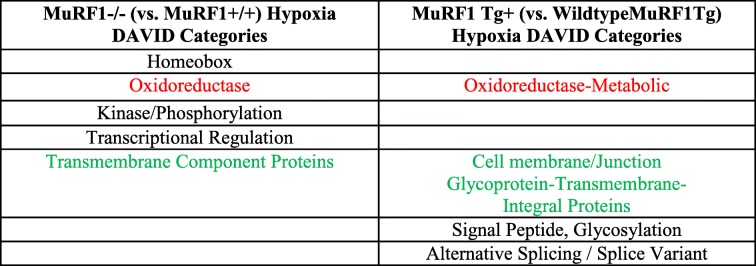
Summary of DAVID functional annotation categories of significant genes. From Figure 3A (MuRF1-/-) and Figure 5A (MuRF1 Tg+) based off differentially expressed genes in Figure 2A and Figure 5A. Matching colors represent related categories found in hearts lacking MuRF1 and hearts with increased cardiomyocyte MuRF1 post-hypoxia challenge compared to wildtype controls

**Fig. 6:**
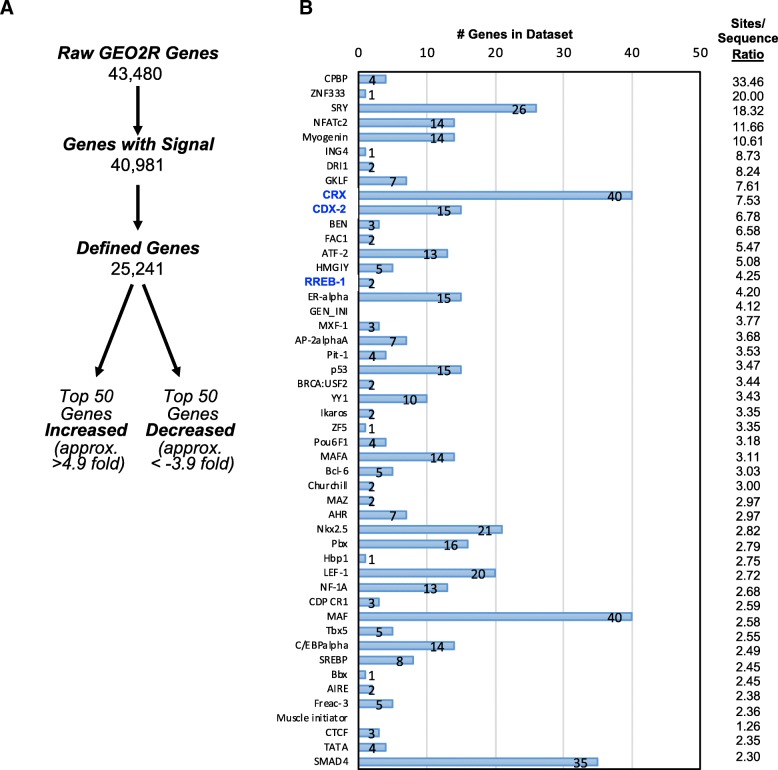
Differentially expressed genes in a rat model of CH-induced pulmonary hypertension and right heart failure. **a.** Summary of how differentially expressed genes in CH-induced rat heart failure resulted in top 50 increased genes (> 4.9-fold) and top 50 decreased genes (< 3.9-fold) compared to normoxia using data from Drake, et al., 2013 [2]. Genes summarized in Additional file 4: Figure S2. **b.** Identification of transcription factors with binding sites of top 50 increased and top 50 decreased genes. The number of genes in the dataset and enrichment (sites/sequence ratio) for each transcription factor is calculated to the right

To give context to our identification of MuRF1-regulated genes in the right ventricle after 3 weeks of CH, we identified a previous study that performed microarray analysis of the right ventricle after CH [2]. We obtained the publicly available data from NCBI GEO to identify the genes differentially expressed in right heart failure induced by CH (Fig. 6). Of the 25,241 defined genes (Fig. 6a), we identified the top 50 genes increased (> 4.9-fold) and top 50 genes decreased (<− 3.9-fold) compared to controls (Additional file 5: Figure S3) and we analyzed these genes using TRANSFAC® FMatch Analysis to identify transcription factors that bind promoters of the significantly altered hypoxia-challenged RV (Fig. 6b). The most enriched transcription factor protein sites included the CREB binding protein (CPBP, 33.46-fold enriched), Zinc Finger Protein 333 (ZNF333, 20-fold enriched), Sex Determining Region Y (SRY) (18.32-fold enriched), and the Nuclear Factor of Activated T-Cells 2 (NFATc2, 11.66-fold enriched) (Fig. 6b). Analysis of the 100 differentially expressed genes from the CH-challenged right ventricle for their relationships in the literature [21] identified 8 groups, including links with PPAR binding protein (PPARBP), regulators of G-protein signaling (RGS5, RGS8, RGS12, RGS14, RGS18, RGS20) and v-ros UR2 sarcoma virus oncogene (ROS1) (Additional file 6: Figure S4). This analysis provides a context of the transcriptional regulation of the RV remodeling that occurs in response to pulmonary hypertension induced right heart failure, demonstrating the transcriptional control involved (Fig. 6b) and the associated connections with proteins in the literature (Additional file 6: Figure S4). When we compared the TRANSFAC Match Analysis identified in the CH-challenged MuRF1−/− right ventricles (vs. wild type) (Fig. 1c) with the MuRF1 Tg + right ventricles (vs. wild type) (Fig. 4a) and hypoxia-induced right heart failure (Fig. 6b), Rreb-1 was identified as a common transcription factor (Table 2). Finally, considerable overlap was seen in Literature Net analysis of the MuRF1−/− significant genes after CH (vs. wild type) (Additional file 4: Figure S2) and the significant genes found in wild-type rat right hearts after CH (Additional file 6: Figure S4), with almost complete overlap (PPARBP, RGS proteins, and ROS1).

**Table 2:**
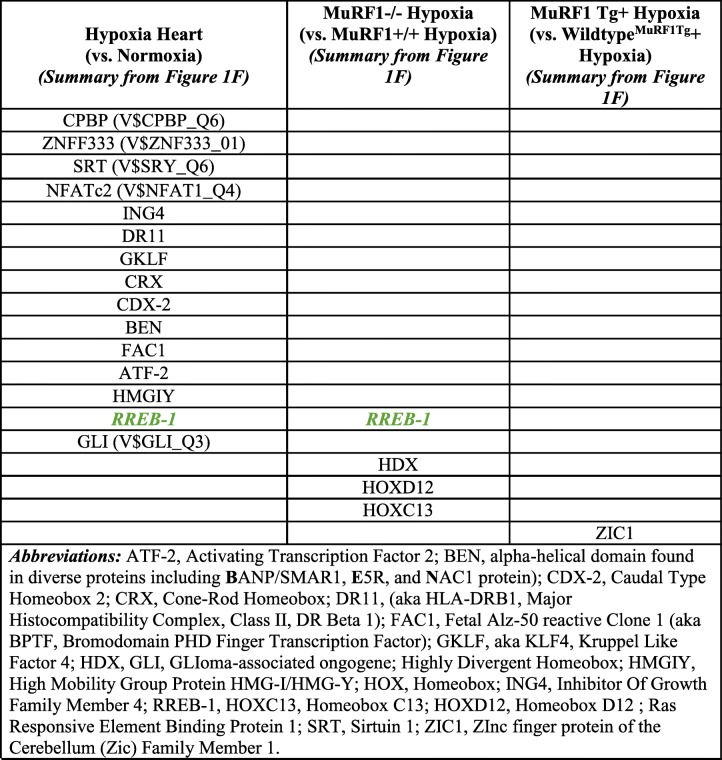
Summary of transcription factors identified in the MuRF1-/- and MuRF1 Tg+ right ventricles (vs. wildtype) by TRANSFAC® Match Analysis. From Figure 2C (MuRF1-/-) and Figure 4C (MuRF1 Tg+) based off differentially expressed genes in Figure 2B and Figure 4B. Matching colors represent related transcription factors identified in hearts lacking MuRF1 and hearts with increased cardiomyocyte MuRF1 post-hypoxia challenge compared to wildtype controls

## Discussion

We recently identified that MuRF1−/− mice were resistant to weeks of CH-induced changes in RV ejection fraction and volume (systolic and diastolic) and exhibited significantly increased skeletal muscle perfusion, consistent with better cardiac function compared to wild-type mice [11]. In the present study, we analyzed the MuRF1−/− and MuRF1 Tg + right ventricle transcriptome by microarray analysis to determine the role of cardiomyocyte MuRF1 in response to CH and resulting pulmonary hypertension. The differentially expressed transcripts in the MuRF1−/− hearts (vs. wild type) were enriched by up to 10-fold for HOXD12, HOXC13 and RREB-1 protein promoter regions (Fig. 1c). The role of HOXD12 and HOXC13 in the heart or muscle has not been previously reported to our knowledge; however, their role in transcription and gene expression (as are other homeodomain proteins) is well-annotated in gene ontology (Fig. 1e). Our analysis of a publicly available dataset from a rat model of CH exposure also identified the Rreb-1 (enriched 4.25-fold) as a transcription factor associated with differentially regulated genes, among other transcription factors (e.g., SRY, CRX, MAF, SMAD4) (Fig. 6b). There are several differences in the experimental design of this previous study that used Sprague-Dawley rats (vs. mice in the present study) and pulmonary artery hypertension induced by a single subcutaneous SU5416 (20 mg/kg) injection and 4 weeks of hypoxia (vs. 3 weeks of hypoxia only) [2]. RREB-1 transcription factor binds specifically to the RAS-response elements (RRE) of gene promoters and acts as a coregulatory factor for ninety-one (91) transcription factors in various tissues [22]. In the heart, the RREB-1 protein notably binds with MEF-2, POU3F2, SRF, PPARalpha/RXRalpha, GR and E2F. While the role of RREB-1 has not been investigated directly in the heart, these seven transcription factors have well-defined roles in heart failure. MEF-2 and SRF, in particular, are known to play critical roles in pathological hypertrophy and are part of the “fetal gene” response in pressure overload-induced heart failure [23]. PPARalpha/RXRalpha have critical roles in regulating fatty acid metabolism in cardiomyocytes and in the context of heart failure have been proposed to play roles in the shift in substrate utilization in heart failure [24].

These findings are notable given what is known about cardiac MuRF1 activity according to the literature. In studies of MuRF1−/− mice challenged with pressure overload-induced hypertrophy via transaortic constriction, MuRF1−/− mice experienced enhanced hypertrophy (cardiomyocyte growth) and decreased SRF-regulated gene expression (fetal genes) [8]. These studies found that MuRF1 immunoprecipitated specifically with SRF and directly inhibited cardiomyocyte SRF activity [8]. Similarly, cardiomyocyte MuRF1 has been reported to interact directly with PPARalpha to inhibit its activity, with MuRF1−/− hearts having a five-fold increase in PPARalpha activity [25]. In these studies, increasing MuRF1 expression demonstrated enhancement of PPARalpha nuclear export, dependent upon specific lysines adjacent to nuclear export sequences found to be mono-ubiquitinated [25]. In additional to MuRF1’s regulation of SRF and PPARalpha, cardiac MuRF1 and MuRF2 have been reported to support transcription factor E2F1 in microarray studies, confirmed by chromatin IP studies in the context of developmental cardiac growth [26, 27]. Since pulmonary (RV) hypertension has numerous parallels with left ventricular pressure overload-induced hypertrophy, the present study may suggest that MuRF1’s regulatory effects on SRF may contribute to the phenotype observed. Taken together, the present study suggests that MuRF1’s regulation of SRF, PPARalpha, and E2F1 may be due to MuRF1’s broader regulation of cotranscription factors such as Rreb-1, given Rreb-1’s established connection with SRF, PPARalpha and E2F1 in other tissues [22].

Beyond the evidence linking MuRF1 to the regulation of transcription, the differentially expressed genes in the MuRF1−/− right ventricle after CH were related to oxidoreductase functions. Oxidoreductases are enzymes that catalyze oxidoreduction reactions and have important roles in aerobic and anaerobic metabolism and the utilization of NAD+/NADH. In the heart, catalase is an oxidoreductase that acts on H_2_O_2_, breaking it down to harmless hydrogen and water [28, 29]. In the present study, the MuRF1−/− right ventricle had several differentially expressed genes related to the oxidoreductase system (*Adh1, Aasdh, Pam, Xdh*) (Fig. 2a). Alcohol dehydrogenase 1 (Adh1) has been identified in the heart to have important antioxidant roles in collaboration with catalase [30]. Expression of aminoadipate-semialdehyde dehydrogenase (*Aasdh*) is highest in the brain, followed by heart and skeletal muscle [31]. In the heart, the membrane bound peptidylglycine alpha-amidating monooxygenase (*Pam*) has been implicated in the shaping of atrial secretory vesicles related to the secretion of pro-ANP [32]. Cell injury from hyperoxia was associated with increased superoxide radicals, which has been associated with xanthine dehydrogenase (*Xdh*) activity in heart [33]. MuRF1−/− right ventricles also had differentially expressed genes (*Araf, Ip6K3, Nmrk2*) associated with kinase activity (Fig. 2a). The role of raf proteins, including A-Raf Proto- Oncogene, Serine/Threonine Kinase (*Araf*), has been described in cardiac hypertrophy and cardiomyocyte survival, with antiapoptotic activity independent of MKK and ERK activities [34, 35]. Inositol hexakisphosphate kinase 3 (*Ip6K3*) has been linked to metabolism and lifespan in mice [36], while nicotinamide riboside kinase 2 (*Nmrk2*) has critical roles in NAD metabolism in mammalian cells [37].

Cardiomyocyte MuRF1 has been indirectly linked to oxidoreductase functions in previous studies. Cardiomyocyte-specific MuRF1 Tg + mice were challenged with an acute cardiac ischemia reperfusion injury both ex vivo and in vitro and demonstrated considerable resistance to injury and functional defects [38]. One mechanism that could explain this protection against acute hypoxia is MuRF1’s inhibition of JNK signaling, mediated by MuRF1’s polyubiquitination of phosphorylated c-Jun (subsequently degraded) [38]. Subsequent studies showed that MuRF1 Tg + heart mitochondria generated significantly less (51–67% less) ROS measured by H_2_O_2_ emission at baseline compared to sibling wild-type control mitochondria [39]. How MuRF1 reduces H_2_O_2_ emission is not known, but the results from the current study may lend some insights.

We utilized the MuRF1 Tg + mice in present study because of the limited redundancy that is known to occur between MuRF1 and other MuRF family members (MuRF2, MuRF3). This redundancy was initially suggested by overlap of some binding partners (e.g., cardiac troponin I [cTnI]) but not others (e.g., myosin light chain 2) [40]. The partial overlap between cardiac MuRF1, MuRF2 and MuRF3 was also seen in nontargeted metabolomics studies of baseline heart tissues [41]. As seen in previous studies of MuRF1 Tg + mice challenged with pressure overload-induced hypertrophy [12], CH resulted in significant decreases in RV weight and ejection fraction, indicating a maladaptive dilated phenotype (heart failure) [11]. In contrast to the MuRF1−/− mice challenged with CH that resulted in improved skeletal muscle perfusion (vs. wild-type mice), MuRF1 Tg + mice exhibited significant decreases in skeletal muscle perfusion [11]. Identification of transcription factors enriched in the differentially expressed genes in the MuRF1 Tg + right ventricle in the present study identified promoter regions that the ZIC1 protein binds (Fig. 4a). The ZIC1 protein is a transcriptional activator described primarily in the development and maturation of the cerebellum [42]. It has also been reported to have a role in shear flow mechanotransduction in osteocytes [43]. Functionally it retains nuclear GLI1 and GLI3 in the cytoplasm by binding the minimal GLI-consensus sequence 5-TGGGTGGTC-3 [44]. Mutations in *Zic* gene family members resulted in congenital heart defects in both humans and mice [45]. The ZIC1 protein has also been shown to act as a protein transcription factor upstream *Pax3* in the induction of embryonic neural crest [46]. While little is known about the ZIC1 protein in the heart, it has been linked to the homeobox domain protein HOXA1 protein. Specifically, Hoxa1 null embryos revealed their regulation of genes in cardiac NC precursors, including Hnf1b, Foxd3, and Zic1 [47]. Sumoylation regulates the nuclear localization and function of closely related ZIC3 protein family members [48, 49].

Hypoxia-induced RV hypertrophy in cardiomyocyte-specific MuRF1 Tg + mice induced gene expression changes functionally linked to oxidoreductase regulation. Of the five differentially expressed genes in the MuRF1 Tg + post-CH related to oxidoreductase regulation (Fig. 5a, far left column), only one had clear roles in the heart. The Delta 4-Desaturase Sphingolipid 2 (*Degs2*) is an enzyme involved in de novo ceramide synthesis. One study reported that ethanol exposure upregulated *Degs* in rats and altered lipids profiles that support beneficial cardioprotective and neuroprotective effects of moderate ethanol consumption [50]. The role of DEGS2 in the heart is unknown. The Cytochrome P450 Family 27 Subfamily B Member 1 (*Cyp27b1*), also known as 25-Hydroxyvitamin D3 1-alpha-hydroxylase, is responsible for the hydroxylation of calcifediol to calcitriol (the bioactive form of vitamin D). Variants in Diacylglycerol Kinase Kappa (*Dgkk*) have been described in association with hypospadias [51–54], but no functions in the heart have been reported. The role of Cyp27b1 in the heart is unknown. Detailed function of the Cytochrome P450 Family 2 Subfamily B Member 13 (*Cyp2b13*) and Arachidonate 12-Lipoxygenase, 12R Type (Alox12b) were unavailable beyond the functional categorization by DAVID (Fig. 5a).

Hypoxia-induced RV hypertrophy in cardiomyocyte-specific MuRF1 Tg + mice also induced gene expression changes functionally linked to alternative splicing/splice variants. Of the 16 differentially expressed genes in the MuRF1 Tg + post-CH related to alternative splicing (Fig. 5a, far right column), four stood out (*Casq1, Myl1, Erbb2ip, Pitx2*). Calsequestrin 1 (Casq1) functions as a calcium buffer in the heart with a role in calcium release by interactions with the ryanodine receptor, having important implications for contractility [55–57]. Myosin Light Chain 1 (*Myl1*) is a GATA4 target gene related to IGF-1 signaling in the context of pathological hypertrophy stimuli [58]. The Erbb2 Interacting Protein (*Erbb2ip*) is present in relatively high levels in the heart and has been found to be down-regulated in heart biopsies from human failing hearts, with a role in attenuated ERK signaling to negatively modulate cardiac hypertrophy experimentally in mice [59]. Mutations in the Paired Like Homeodomain 2 (*Pitx2*) gene in the heart have been seen in the Tetralogy of Fallot and atrial fibrillation in patients [60–64]. Interestingly, Pitx2 was the only gene in common between the set of 66 differentially expressed genes in the MuRF1−/− hearts and the set of 45 differentially expressed genes in the MuRF1 Tg + hearts. Pitx2 expression was induced in RV in mice lacking MuRF1 and was repressed in response to MuRF1 overexpression, suggesting an exquisite sensitivity of this gene to levels of MuRF1.The Fatty Acid Synthase (*FASN* aka *C93*) gene was associated with lipid metabolism reprogramming in arrhythmogenic RV cardiomyopathy [65], while hypoxia has been associated with FASN-dependent free fatty acid production [66–68]. Lastly, the RAB3 GTPase Activating Protein Catalytic Subunit 1 (*Rab3gap1*) has been associated with cardiovascular risk (total cholesterol and HDL) by GWAS association in Framingham Heart Studies [69], with *Rab3gap1* loci having associations with an increased risk of SCD [70]. The remaining differentially expressed genes in MuRF1 Tg + hearts after CH exposure associated with alternative splicing (*Ptpru, Svopl, Syt16, Ccp110, Jakmip3, Kansl1l*) have not been functionally defined in heart and/or cardiac disease to our knowledge.

## Conclusions

Analysis of the top differentially expressed transcripts in the MuRF1−/− right ventricle after CH revealed genes involved in the regulation of transcription and oxidoreductase functions. Bioinformatic analysis suggested that the differentially expressed genes in MuRF1−/− right ventricle tissue were linked to HOX (homeobox) transcription factors and Rreb-1. Analysis of the top differentially expressed transcripts in the cardiomyocyte-specific MuRF1 Tg + − right ventricle after CH revealed genes involved in the regulation of oxidoreductase-metabolic, glycoprotein-transmembrane and alternative splicing functions. Together, the MuRF1−/− and MuRF1 Tg + results have common functional annotations related to oxidoreductase (including antioxidant functions) and transmembrane component functions (Table 1). These studies reveal potential indirect effects of MuRF1 on known substrates such as SRF and PPARalpha by regulation of Rreb-1. The results also give insight into potential mechanisms by which MuRF1 regulates antioxidant systems by transcriptionally regulating genes related to oxidoreductase functions.

## Additional files

Additional file 1: **Table S1.** Differentially expressed genes in MuRF1−/− mice challenged with hypoxia (XLSX 59 kb)

Additional file 2: **Table S2.** Differentially expressed genes in MuRF1 Tg + mice challenged with hypoxia (XLSX 22 kb)

Additional file 3: **Figure S1.** Validation of differential expressed genes in MuRF1−/− hearts. Reverse transcriptase quantitative PCR analysis of MuRF1−/− hearts of differentially expressed genes Gbp3 and Cxcl9 mRNA in the right ventricle. (PDF 26 kb)

Additional file 4: **Figure S2.** Literature Net analysis of differentially expressed genes in MuRF1 Tg + right ventricles after chronic hypoxia challenge compared to wildtype controls. Analysis of the top 28 genes increased (> 3 fold) and top 18 genes decreased (<− 3 fold) listed in Fig. 3b using Literature Net on Duke Gather (http://changlab.uth.tmc.edu/gather/gather.py). (PDF 24 kb)

Additional file 5: **Figure S3.** Top differentially expressed genes by microarray analysis of right ventricle tissue in rats challenged with pulmonary hypertension-induced right heart failure. A. Top 50 genes increased compared to normoxia controls. B. Top 50 genes decreased compared to normoxia controls. Data downloaded from Gene Expression Omnibus (GEO) GEO2R interface including the 8 normoxia right ventricle replicates (6 biological replicates) and 12 hypoxia right ventricle replicates (6 biological replicates). 43,480 data points were downloaded, with 25.241 named. The top 50 genes here were used in further analysis of related transcirption factors. Data published in Drake, et al., Physiol Genomics 2013 45(12):449–61. (PDF 52 kb)

Additional file 6: **Figure S4.** Literature Net analysis of differentially expressed genes in right ventricles after chronic hypoxia challenge compared to normoxic controls using previously published microarray data. Analysis of the top 50 genes increased (> 4.9 fold) and top 50 genes decreased (<− 3.9 fold) listed in Additional file 4: Figure S2 using Literature Net on Duke Gather (http://changlab.uth.tmc.edu/gather/gather.py). Publicly available data obtained from NCBI GEO published in Drake, et al., Physiol Genomics 2013 45(12):449–61, as described in the materials and methods. (PDF 24 kb)

## Abbreviations

ATF-2: Activating Transcription Factor 2
Atp2b2: ATPase Plasma Membrane Ca2+ Transporting 2
BEN: Alpha-helical domain found in diverse proteins including BANP/SMAR1, E5R, and NAC1 protein)
Casq1: Calsequestrin 1
CDX-2: Caudal Type Homeobox 2; CPBP, (aka Kruppel Like Factor 6, KLF6)
CRX: Cone-Rod Homeobox
DR11: (Aka HLA-DRB1, Major Histocompatibility Complex, Class II, DR Beta 1)
Elovl7: ELOVL Fatty Acid Elongase 7
FAC1: Fetal Alz-50 reactive Clone 1 (aka BPTF, Bromodomain PHD Finger Transcription Factor)
GKLF: Aka KLF4, Kruppel Like Factor 4
GLI: GLIoma-associated ongogene homolog 1 (Zinc Finger Protein)
HDX: Highly Divergent Homeobox
HMGIY: High Mobility Group Protein HMG-I/HMG-Y
HOX: Homeobox
ING4: Inhibitor Of Growth Family Member 4
MuRF1 Tg +: MuRF1 transgenic
MuRF1: Muscle ring finger-1
MuRF2: Muscle ring finger-2
Myl1: Myosin Light Chain 1
NFATc2: Nuclear Factor of Activated T-cells 2
Rreb-1: Ras Responsive Element Binding Protein 1
SRT: Sirtuin 1
Zic1: Zinc finger protein of the Cerebellum (Zic) Family Member 1
ZNFF333: Zinc Finger Protein 333
