# Supplementary material for: Muscle-specific regulation of right ventricular transcriptional responses to chronic hypoxia-induced hypertrophy by the muscle ring finger-1 (MuRF1) ubiquitin ligase in mice
Source: BMC Med Genet. 2018 Sep 21;19:175. doi: 10.1186/s12881-018-0670-1 (PMC6150973; doi:10.1186/s12881-018-0670-1)
Supplement: Supplementary file 3 — Figure S1. Validation of differential expressed genes in MuRF1−/− hearts. Reverse transcriptase quantitative PCR analysis of MuRF1−/− hearts of differentially expressed genes Gbp3 and Cxcl9 mRNA in the right ventricle. (PDF 26 kb) [file 12881_2018_670_MOESM3_ESM.pdf]

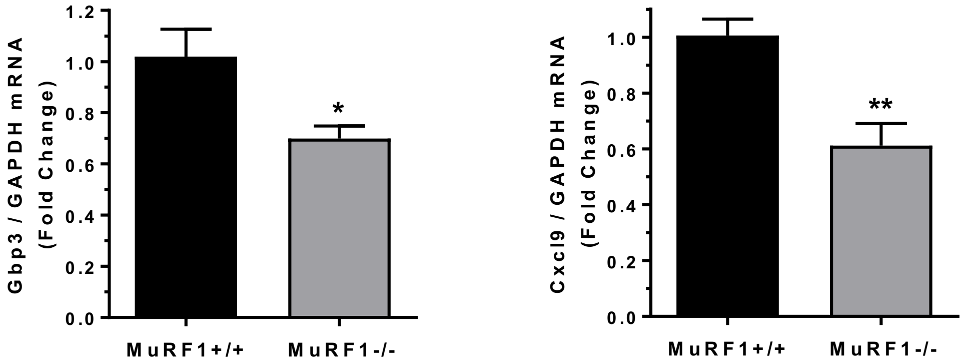

Supplemental Figure 1. Validation of differential expressed genes in MuRF1-/- hearts. Reverse transcriptase quantitative PCR analysis of MuRF1-/- hearts of differentially expressed genes *Gbp3* and *Cxcl9* mRNA in the right ventricle.
