# Supplementary material for: Muscle-specific regulation of right ventricular transcriptional responses to chronic hypoxia-induced hypertrophy by the muscle ring finger-1 (MuRF1) ubiquitin ligase in mice
Source: BMC Med Genet. 2018 Sep 21;19:175. doi: 10.1186/s12881-018-0670-1 (PMC6150973; doi:10.1186/s12881-018-0670-1)
Supplement: Supplementary file 4 — Figure S2. Literature Net analysis of differentially expressed genes in MuRF1 Tg + right ventricles after chronic hypoxia challenge compared to wildtype controls. Analysis of the top 28 genes increased (> 3 fold) and top 18 genes decreased (<− 3 fold) listed in Fig. 3b using Literature Net on Duke Gather (http://changlab.uth.tmc.edu/gather/gather.py). (PDF 24 kb) [file 12881_2018_670_MOESM4_ESM.pdf]

Literature Net

	#Genes	p value	Bayes Factor
**1. Ebaf2: endometrial bleeding associated factor**<br>**Genes:** *Pitx2*	1	0.0003	6
**2. PPARBP: PPAR binding protein**<br>**Genes:** *CYP27B1[H] TNMD[H]*	2	0.0003	6
**3. RGS14: regulator of G-protein signaling 14**<br>**Genes:** *PITX2[H]*	1	0.002	5
**4. RGS12: regulator of G-protein signaling 12**<br>**Genes:** *PITX2[H]*	1	0.002	5
**5. RGS18: regulator of G-protein signaling 18**<br>**Genes:** *PITX2[H]*	1	0.002	5
**6. RGS5: regulator of G-protein signaling 5**<br>**Genes:** *PITX2[H]*	1	0.002	5
**7. RGS8: regulator of G-protein signaling 8**<br>**Genes:** *PITX2[H]*	1	0.002	5
**8. RGS20: regulator of G-protein signaling 20**<br>**Genes:** *PITX2[H]*	1	0.002	5
**9. ROS1: v-ros UR2 sarcoma virus oncogene homolog 1**<br>**Genes:** *CYP27B1[H] PTPRU[H] TNMD[H]*	3	0.003	5
**10. Il22ra1: interleukin 22 receptor, alpha 1**<br>**Genes:** *Il22*	1	0.003	4
**11. Ef1alpha48D: Elongation factor 148D**<br>**Genes:** *Frq[H] Mlc1[H]*	2	0.004	4
**12. PCSK1: proprotein convertase subtilisin/kexin type 1**<br>**Genes:** *PTPRU[H] TNMD[H]*	2	0.004	4

Supplemental Figure 2. Literature Net analysis of differentially expressed genes in MuRF1 Tg+ right ventricles after chronic hypoxia challenge compared to wildtype controls. Analysis of the top 28 genes increased (>3 fold) and top 18 genes decreased (<-3 fold) listed in Figure 3B using Literature Net on Duke Gather (http://changlab.uth.tmc.edu/gather/gather.py)
