# Supplementary material for: Muscle-specific regulation of right ventricular transcriptional responses to chronic hypoxia-induced hypertrophy by the muscle ring finger-1 (MuRF1) ubiquitin ligase in mice
Source: BMC Med Genet. 2018 Sep 21;19:175. doi: 10.1186/s12881-018-0670-1 (PMC6150973; doi:10.1186/s12881-018-0670-1)
Supplement: Supplementary file 5 — Figure S3. Top differentially expressed genes by microarray analysis of right ventricle tissue in rats challenged with pulmonary hypertension-induced right heart failure. A. Top 50 genes increased compared to normoxia controls. B. Top 50 genes decreased compared to normoxia controls. Data downloaded from Gene Expression Omnibus (GEO) GEO2R interface including the 8 normoxia right ventricle replicates (6 biological replicates) and 12 hypoxia right ventricle replicates (6 biological replicates). 43,480 data points were downloaded, with 25.241 named. The top 50 genes here were used in further analysis of related transcirption factors. Data published in Drake, et al., Physiol Genomics 2013 45(12):449–61. (PDF 52 kb) [file 12881_2018_670_MOESM5_ESM.pdf]

Hypoxia-induced Right Heart Failure (vs. Normoxia controls)

A *Top 50 Genes* ***Increased*** *(>4.9 fold)*

Gene.symbol	logFC
Acbd3	7.7399098
Anks6	7.4512372
Lyplal1	7.3525136
Syt9	7.3075573
Olr601	7.2756361
Olr665	7.1268722
Afm	6.7279746
Nr3c1	6.6294987
Cmtm3	6.5814714
Ankle2	6.529752
Tbr1	6.4051254
Prss21	6.3764999
Cyp1a1	6.356943
Olr1435	6.3283155
Olr456	6.2585462
Slc46a2	6.240707
Chodl	6.2322086
Asip	6.1616372
Ifnk	5.903773
Abcb1a	5.8294556
Hmmr	5.780133
Kif3c	5.6894731
Cabp5	5.6881792
Akap11	5.6510136
Vom2r65	5.6499162
Slc22a8	5.6490462
Micu3	5.6447986
Muc3	5.6226814
St18	5.6049835
RGD1563222	5.5372526
Ddr1	5.4703543
Hcrtr2	5.3809926
Nipsnap3b	5.3559893
Olr1637	5.3303087
Kcnf1	5.311148
Olr1405	5.1979302
RGD1309995	5.1728245
Zfat	5.1593694
Etnk1	5.1479151
Mapk6	5.1012051
Gpx2	5.08466
RGD1308544	5.0699919
Lrrc6	5.0571202
Aqp4	5.0495376
Olr1739	5.0260437
Papd4	5.0139808
Serpinb8	5.0060356
Hspa12a	5.0039469
P2rx2	4.9947203
Olr655	4.9889469

B *Top 50 Genes* ***Decreased*** *(< -3.9 fold)*

Gene.symbol	logFC
Glra4	-3.9443876
Ryr2	-3.9565372
Ppargc1a	-3.9571665
Slc30a3	-3.9704991
Pla2g5	-3.9990494
Kif5a	-4.0163281
Syt1	-4.0505071
Apcs	-4.0612704
Myzap	-4.0772834
Mpo	-4.0889497
Dab1	-4.1061027
Ophn1	-4.1318639
Cxxc4	-4.1365402
Zfp362	-4.1560058
Lynx1	-4.1604026
Wdr93	-4.2234658
Scgn	-4.2503407
Olr357	-4.2533781
Rhpn2	-4.2538903
Nmnat1	-4.2707181
Gap43	-4.2881502
Olr1273	-4.3011472
Hmgcs2	-4.3081376
Cartpt	-4.3303303
Slc14a1	-4.3336196
Mcmdc2	-4.3498964
Slc22a3	-4.450574
Ptprt	-4.4890443
Ncbp1	-4.5025758
Sun2	-4.5250063
Tcf7l1	-4.6203004
Ar	-4.6256036
Dcdc2	-4.6454874
Drd1	-4.6749575
Kcng2	-4.6806576
Ebp	-4.7454553
Arhgap36	-4.9438548
Gng12	-4.9697361
Utp20	-5.0410298
Agpat3	-5.1121833
Ryr2	-5.2709372
Pcdh12	-5.2869936
Cartpt	-5.3539069
Sox6	-5.3618295
Glyat	-5.4572406
Fhl5	-5.5295489
Acvr1c	-6.2015081
Pja2	-6.4006704
Poteg	-6.694906
Tsga10	-7.7642788

Supplemental Figure 3. Top differentially expressed genes by microarray analysis of right ventricle tissue in rats challenged with pulmonary hypertension-induced right heart failure. A. Top 50 genes increased compared to normoxia controls. **B.** Top 50 genes decreased compared to normoxia controls. Data downloaded from Gene Expression Omnibus (GEO) GEO2R interface including the 8 normoxia right ventricle replicates (6 biological replicates) and 12 hypoxia right ventricle replicates (6 biological replicates). 43,480 data points were downloaded, with 25.241 named. The top 50 genes here were used in further analysis of related transcirption factors. Data published in Drake, et al., *Physiol Genomics* 2013 45(12):449-61.
