# Supplementary material for: Muscle-specific regulation of right ventricular transcriptional responses to chronic hypoxia-induced hypertrophy by the muscle ring finger-1 (MuRF1) ubiquitin ligase in mice
Source: BMC Med Genet. 2018 Sep 21;19:175. doi: 10.1186/s12881-018-0670-1 (PMC6150973; doi:10.1186/s12881-018-0670-1)
Supplement: Supplementary file 6 — Figure S4. Literature Net analysis of differentially expressed genes in right ventricles after chronic hypoxia challenge compared to normoxic controls using previously published microarray data. Analysis of the top 50 genes increased (> 4.9 fold) and top 50 genes decreased (<− 3.9 fold) listed in Additional file 4: Figure S2 using Literature Net on Duke Gather (http://changlab.uth.tmc.edu/gather/gather.py). Publicly available data obtained from NCBI GEO published in Drake, et al., Physiol Genomics 2013 45(12):449–61, as described in the materials and methods. (PDF 24 kb) [file 12881_2018_670_MOESM6_ESM.pdf]

Literature Net

	#Genes	p value	Bayes Factor
**PPARBP: PPAR binding protein**<br>**Genes:** *CYP27B1 TNMD*	2	0.0001	6
**RGS14: regulator of G-protein signaling 14**<br>**Genes:** *PITX2*	1	0.001	5
**RGS12: regulator of G-protein signaling 12**<br>**Genes:** *PITX2*	1	0.001	5
**RGS18: regulator of G-protein signaling 18**<br>**Genes:** *PITX2*	1	0.001	5
**RGS5: regulator of G-protein signaling 5**<br>**Genes:** *PITX2*	1	0.001	5
**RGS8: regulator of G-protein signaling 8**<br>**Genes:** *PITX2*	1	0.001	5
**RGS20: regulator of G-protein signaling 20**<br>**Genes:** *PITX2*	1	0.001	5
**ROS1: v-ros UR2 sarcoma virus oncogene**<br>**Genes:** *CYP27B1 PTPRU TNMD*	3	0.001	5

Supplemental Figure 4. Literature Net analysis of differentially expressed genes in right ventricles after chronic hypoxia challenge compared to normoxic controls using previously published microarray data. Analysis of the top 50 genes increased (>4.9 fold) and top 50 genes decreased (<-3.9 fold) listed in Supplemental Figure 2 using Literature Net on Duke Gather (http://changlab.uth.tmc.edu/gather/gather.py). Publicly available data obtained from NCBI GEO published in Drake, et al., *Physiol Genomics* 2013 45(12):449-61, as described in the materials and methods.
